# The endophytic bacterium *Sphingomonas* SaMR12 alleviates Cd stress in oilseed rape through regulation of the GSH-AsA cycle and antioxidative enzymes

**DOI:** 10.1186/s12870-020-2273-1

**Published:** 2020-02-06

**Authors:** Qiong Wang, Chaofeng Ge, Shun’an Xu, Yingjie Wu, Zulfiqar Ali Sahito, Luyao Ma, Fengshan Pan, Qiyao Zhou, Lukuan Huang, Ying Feng, Xiaoe Yang

**Affiliations:** 1 MOE Key Laboratory of Environment Remediation and Ecological Health, College of Environmental and Resource Sciences, Zhejiang University, Xihu District Yuhangtang Road No. 866, Hangzhou, 310058 People’s Republic of China; 2 Hailiang Group Co., Ltd., Hangzhou, 310058 China

**Keywords:** Cadmium, *Sedum alfredii* Hance, Plant growth-promoting bacteria (PGPB), Non-host plants, Antioxidant, Gene expression

## Abstract

**Background:**

Microbes isolated from hyperaccumulating plants have been reported to be effective in achieving higher phytoextraction efficiency. The plant growth-promoting bacteria (PGPB) SaMR12 from the cadmium (Cd)/zinc hyperaccumulator *Sedum alfredii* Hance could promote the growth of a non-host plant, oilseed rape, under Cd stress. However, the effect of SaMR12 on *Brasscia juncea* antioxidative response under Cd exposure was still unclear.

**Results:**

A hydroponic experiment was conducted to study the effects of *Sphingomonas* SaMR12 on its non-host plant *Brassica juncea* (L.) Czern. under four different Cd treatments. The results showed that SaMR12 could colonize and aggregate in the roots and then move to the shoots. SaMR12 inoculation promoted plant growth by up to 71% in aboveground biomass and 81% in root biomass over that of the non-inoculated plants. SaMR12-inoculated plants significantly enhanced root Cd accumulation in the 10 and 20 μM Cd treatments, with 1.72- and 0.86-fold increases, respectively, over that of the non-inoculated plants. SaMR12 inoculation not only decreased shoot hydrogen peroxide (H_2_O_2_) content by up to 38% and malondialdehyde (MDA) content by up to 60% but also reduced proline content by 7–30% in shoots and 17–32% in roots compared to the levels in non-inoculated plants. Additionally, SaMR12 inoculation promoted the activities of superoxide dismutase (SOD), peroxidase (POD), catalase (CAT), and ascorbate peroxidase (APX) and facilitated the relative gene expression levels of dehydroascorbate reductase (*DHAR*) and glutathione reductase (*GR*) involved in the glutathione (GSH)-ascorbic acid (AsA) cycle.

**Conclusions:**

The results demonstrated that, under Cd stress, SaMR12 inoculation could activate the antioxidative response of *B. juncea* by decreasing the concentrations of H_2_O_2_, MDA and proline, increasing the activities of antioxidative enzymes, and regulating the GSH-AsA cycle. These results provide a theoretical foundation for the potential application of hyperaccumulator endophytic bacteria as remediating agents to improve heavy metal tolerance within non-host plant species, which could further improve phytoextraction efficiency.

**Graphical abstract:**

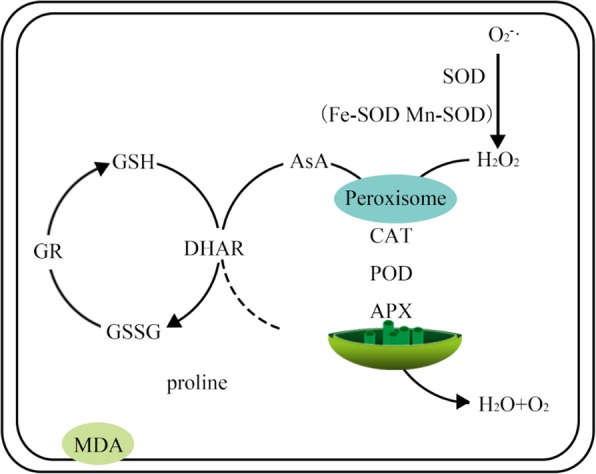

## Background

When metal concentrations in the environment exceed the maximum tolerance of plants, it affects plant physiological and biochemical functions and further results in plant growth retardation and yield degradation [44]. Cadmium (Cd), as a non-nutritive heavy metal (HM), causes plant growth inhibition and affects nutrient uptake and homeostasis even in very small quantities [49, 26]. Prolonged Cd exposure adversely affects several metabolic processes and leads to phytotoxicity, which is caused by limited photosynthetic activity and enzyme activity and the generation of reactive oxygen species (ROS) such as superoxide radicals (O_2_•^−^), hydroxyl (•OH), and hydrogen peroxide (H_2_O_2_) [2, 3, 47, 50, 52]. Moreover, high levels of ROS have damaging effects on plant cellular components, such as membranes, nucleic acids, and chloroplast pigments, which result in plant lipid peroxidation [54].

Plants, including *Brassica juncea* (L.) Czern., have evolved a series of protective and damage repair systems to react to and minimize the effect of oxidative stress. These defence systems are mainly antioxidants, such as glutathione (GSH) and ascorbic acid (AsA) [38, 50], and antioxidant enzymes, including superoxide dismutase (SOD), catalase (CAT), ascorbate peroxidase (APX) and glutathione reductase (GR) [40]. Meanwhile, the genes encoding their respective antioxidants, such as *GR, SOD, APX,* and *CAT,* showed higher expression levels in response to Cd stress, acting as an adaptive response that could alleviate and minimize oxidative damage [15, 30, 39].

Phytoremediation is an eco-friendly method for pollutant removal that uses living plants to eliminate HMs and has been considered a highly promising technology for remediation of polluted sites ([46, 59]). However, in recent years, because of the lower biomass of hyperaccumulators and the phytotoxic effects of high HM concentrations on normal plants, improving phytoremediation efficiency has gained much attention [35]. With its characteristics of rapid growth, massive biomass and moderate accumulation of Cd, *B. juncea* has been considered one of the most promising plants for the phytoremediation of Cd-contaminated farmlands [22]. In addition, plant-microbe interactions in soil have led to the improvement of phytoextraction efficiency due to the potential role of microorganisms in eliminating the HM-induced toxicity and their positive effect on plant growth promotion in metal-contaminated soils [28, 34]. Previous studies have shown that plant growth-promoting bacteria (PGPB) not only reduce biotic or abiotic stress but also promote plant growth [33]. *Bacillus* spp. alleviated lead (Pb) and arsenic (As) stress in rice by reducing lipid peroxidation and increasing amylase and protease levels to promote plant growth in heavy metal-polluted soil ([43]). Several studies documented that endophytic bacteria associated with plant growth promotion, such as *Bacillus licheniformis,* enhanced copper (Cu), zinc (Zn), chromium (Cr), Cd and Pb accumulation and distribution in plants grown in heavy metal-contaminated soil, which led to reduced levels of toxic metals in the soil (Brunetti et al. 2012). The endophytic bacterium *Pseudomonas fluorescens* Sasm05 promoted *Sedum alfredii* Hance growth and enhanced Cd accumulation in response to Cd stress [13].

The PGPB *Sphingomonas* SaMR12, first isolated from the surface-sterilized root of a Chinese native Cd/Zn hyperaccumulator *S. alfredii* [60, 61], was shown to promote plant growth, protect *S. alfredii* roots from Cd damage and alleviate ROS damage by decreasing H_2_O_2_ and O_2_•^−^ [11, 12, 63]. In a pot experiment, SaMR12 showed a positive effect on *Brassica napus* growth, plant Cd uptake and Cd translocation to the leaves [42]. However, the mechanisms of its promotion effect have not yet been elucidated. Therefore, we inoculated SaMR12 into *B. juncea* under hydroponic culture conditions and investigated its effects on 1) plant growth and Cd uptake at different Cd levels; 2) the responses of antioxidant enzymes and the GSH-AsA cycle; and 3) the expression levels of the responsive genes.

## Results

### Colonization pattern of SaMR12 in oilseed rape

**Fig. 1:**
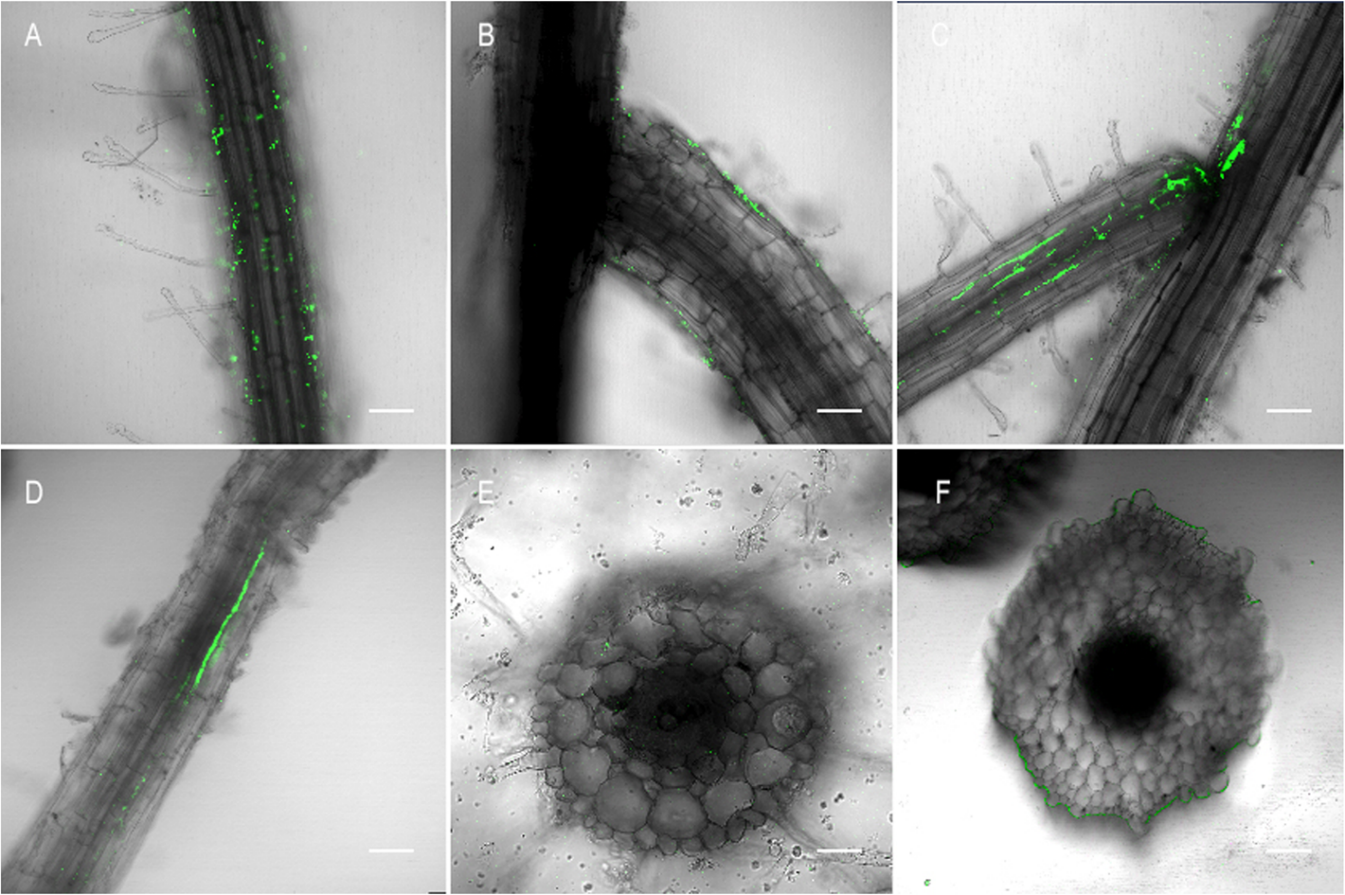
Observation of GFP-tagged *Sphingomonas* sp. SaMR12 colonization pattern with a laser scanning confocal microscope (LSCM). SaMR12 bacteria were cultured in LB medium and inoculated into plants. Roots cut off at different sampling times, (**a**) 2 h, (**b**) 12 h, (**c**) 24 h, and (**d**) 4 d and (**e**) a root section and (**f**) a stem section of *Brassica juncea* (**l**.) Czern. were captured accordingly. The images present bacterial GFP fluorescence in green. SaMR12 colonize and aggregated on the surface of roots and root junction sites, followed by further transportation to aboveground tissues. Bars = 50 μm

Roots were cut into 5 mm pieces at 2 h, 12 h, 24 h and 4 d after SaMR12 inoculation to observe the bacterial colonization pattern using a laser scanning confocal microscope (CLSM) (Fig. 1). Roots were surrounded with single cells at the initial 2 h (Fig. 1a). From 2 h–12 h, some cells successfully invaded the root, mainly through the lateral primordia (Fig. 1b). At 24 h, many more cells had invaded the root and assembled in the root (Fig. 1c). The gathered cells were likely transported along the intercellular space of the plant. After 4 d, the cells were stably colonized inside the root (Fig. 1d). Further observation of root and stem cross-section showed that the bacteria were increasingly present in the roots and were transported to the shoots (Fig. 1e, f).

### Effect of SaMR12 on plant growth and cd accumulation

**Fig. 2:**
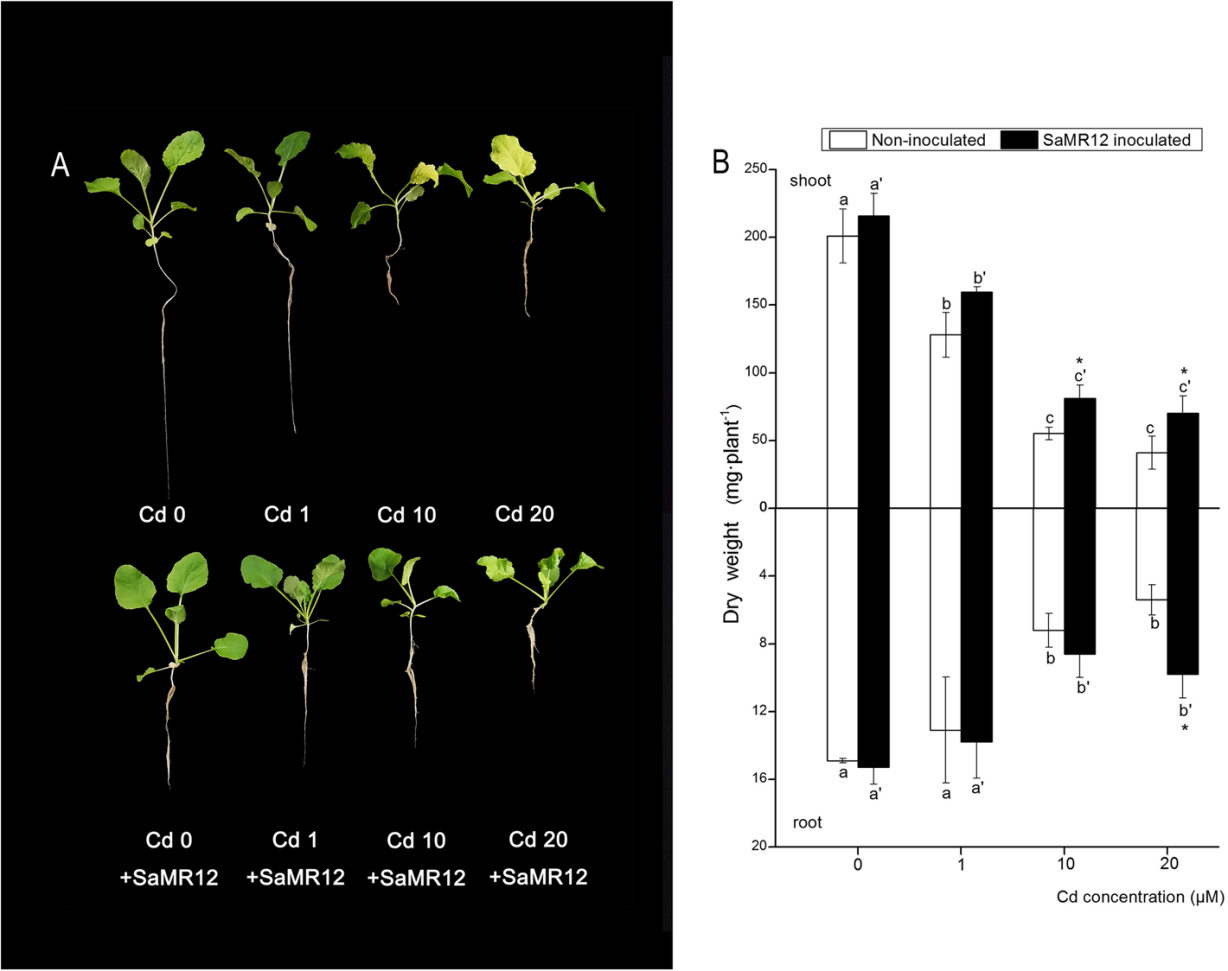
The effect of *Sphingomonas* SaMR12 on (**a**) plant growth and (**b**) plant biomass. The white column, non-inoculated plants; the black column, SaMR12-inoculated plants. Plant samples were harvested after 7 d of treatment. The vertical line on each bar shows the standard deviation (*n* = 3). The different letters on the error bars indicate significant differences among treatments at *p* < 0.05 in shoot and root, respectively. The same letters above the bars indicate no difference between the treatments at *p* < 0.05. Asterisk (*) and double asterisks (**) indicate *p* < 0.05 and *p* < 0.01, respectively, for Student’s t-test carried out between SaMR12 non-inoculated and SaMR12-inoculated samples

Cd exposure decreased plant biomass in both shoot and root parts as the Cd application level increased (Fig. 2). Cd affected root development and depressed plant growth (Fig. 2a). Inoculation with endophytic SaMR12 significantly enhanced the shoot biomass at 10 and 20 μM Cd treatment, with 0.46- and 0.71-fold increases, respectively, compared to their respective non-inoculated treatments (Fig. 2b). Inoculation with SaMR12 enhanced root biomass, but significant improvements were only detected under 20 μM Cd treatment with a 0.81-fold increase over that of the non-inoculated treatment (Fig. 2b).

**Fig. 3:**
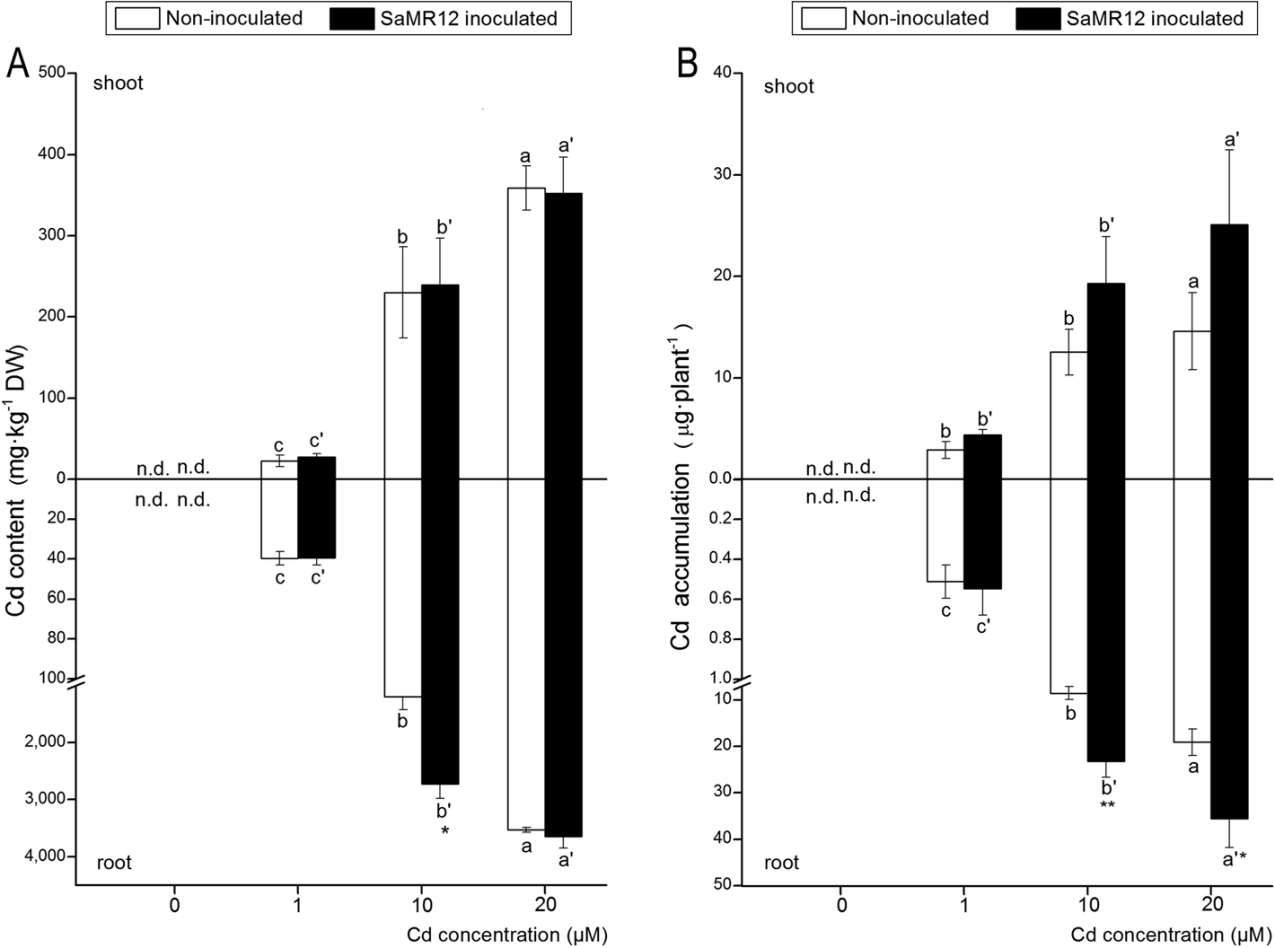
The effect of *Sphingomonas* SaMR12 on (**a**) Cd concentration and (**b**) Cd accumulation in both shoots and roots. And n.d. stands for not detected. The white column, non-inoculated plants; the black column, SaMR12-inoculated plants. Plant samples were harvested after 7 d of treatment. The vertical line on each bar shows the standard deviation (*n* = 3). The different letters on the error bars indicate significant differences among treatments at *p* < 0.05 in shoot and root, respectively. The same letters above the bars indicate no difference between the treatments at *p* < 0.05. Asterisk (*) and double asterisks (**) indicate *p* < 0.05 and *p* < 0.01, respectively, for Student’s t-test carried out between SaMR12 non-inoculated and SaMR12-inoculated samples

The Cd content in both the aerial and underground parts significantly increased with increasing Cd levels (Fig. 3a). Inoculation with SaMR12 significantly promoted root Cd content at 10 μM Cd treatment, with a 1.29-fold increase over that of the non-inoculated treatment (Fig. 3a). As the Cd treatment level increased, Cd accumulation in both the aerial and underground parts significantly increased among the different Cd level treatments (Fig. 3b). SaMR12 inoculation increased Cd accumulation in the shoot part and showed a significant promotion effect on root Cd accumulation at 10 and 20 μM Cd treatment, with increases of 172 and 86%, respectively, over those of the non-inoculated treatments (Fig. 3b).

### Effect of SaMR12 on H_2_O_2_ content, MDA content and O_2_•^−^ levels

**Fig. 4:**
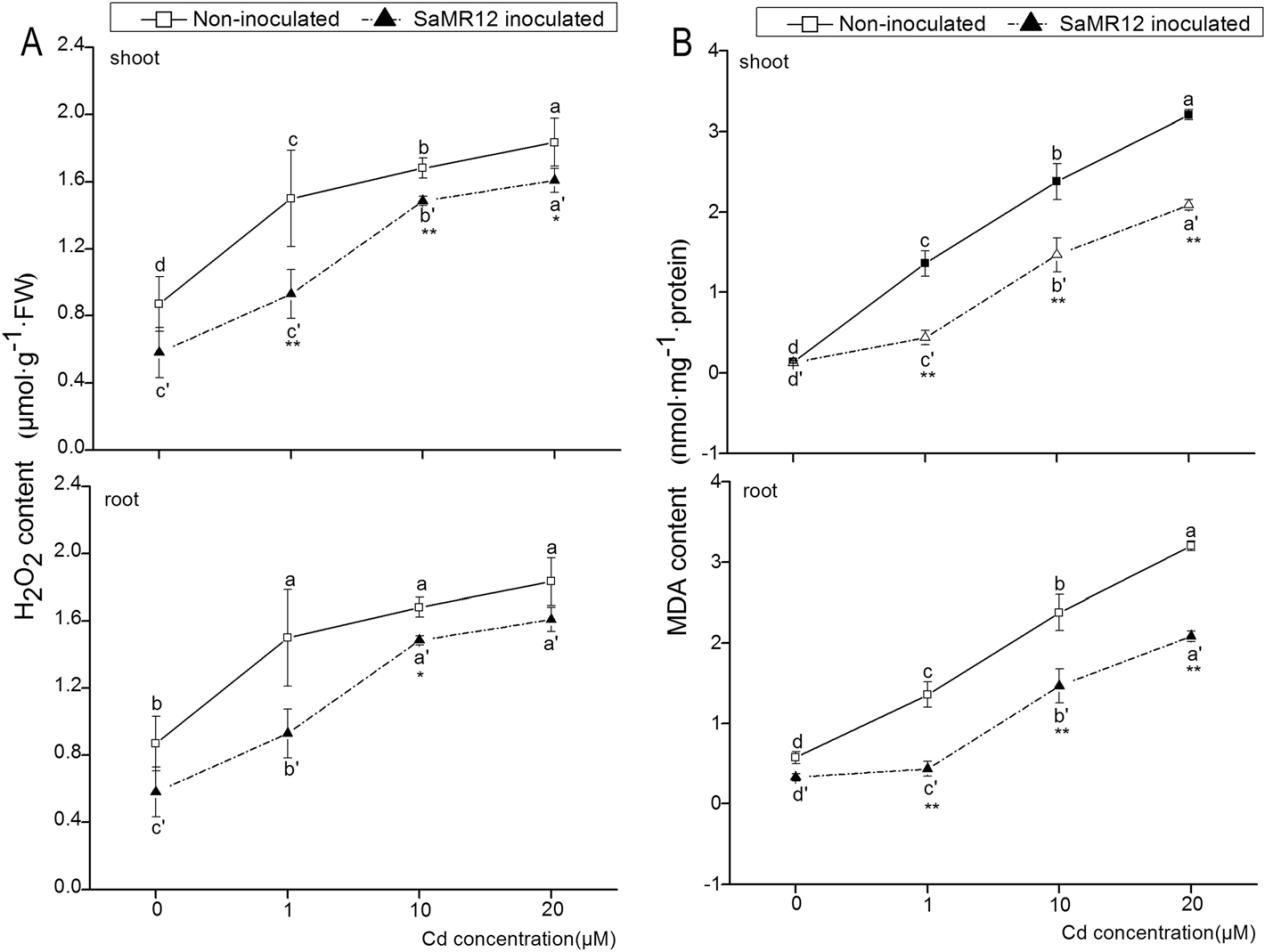
The effect of *Sphingomonas* SaMR12 on (**a**) H_2_O_2_ content, (**b**) MDA content in both shoots and roots. The solid line, non-inoculated plants; the dashed line, SaMR12-inoculated plants. Plant samples were harvested after 7 d of treatment. The vertical line on each bar shows the standard deviation (*n* = 3). The different letters on the error bars indicate significant differences among treatments at *p* < 0.05 in the shoot and root, respectively. The same letters above the bars indicate no difference between the treatments at *p* < 0.05. Asterisk (*) and double asterisks (**) indicate *p* < 0.05 and *p* < 0.01, respectively, for Student’s t-test carried out between SaMR12 non-inoculated and SaMR12-inoculated samples

Cd treatments activated H_2_O_2_ content increases in both the shoot and the root and caused a significantly higher H_2_O_2_ content compared with that of the 0 μM Cd treatment (Fig. 4a). Compared with that in the non-inoculated plants, SaMR12 inoculation significantly decreased shoot H_2_O_2_ content at all Cd treatment levels by 33, 38, 11 and 12%, respectively. In the underground parts, a lower root H_2_O_2_ content at the *p* < 0.05 level was observed only in the 10 μM Cd treatment (Fig. 4a).

**Fig. 5:**
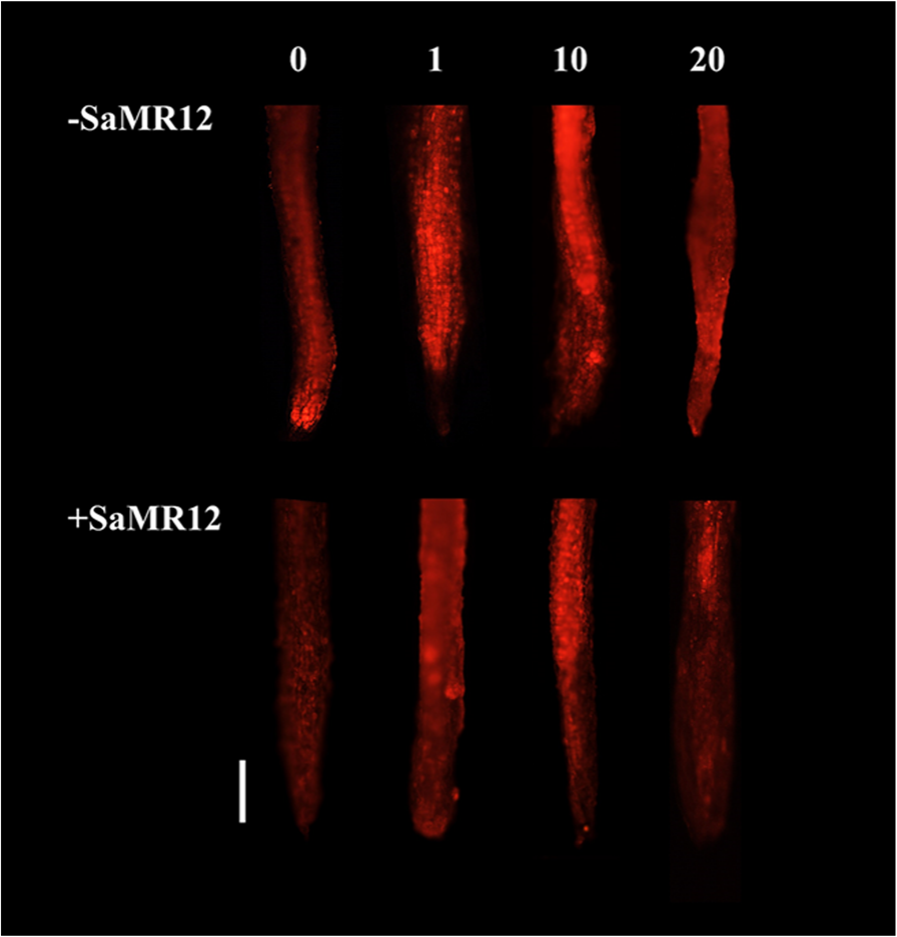
Fluorescence imaging of O_2_•^−^ in root tips using dihydroethidium. *Sphingomonas* SaMR12 alleviated the abiotic stress induced by Cd treatment. Bar = 100 μm

After 7 d of treatment, the MDA content of all plants significantly increased with increasing Cd treatment levels and reached the highest level after 20 μM Cd treatment in both the aerial and root parts (Fig. 4b). At the 0 μM Cd treatment, the MDA level was the lowest in both SaMR12-inoculated and non-inoculated plants, and inoculation with SaMR12 showed a very small effect on the MDA content. When Cd was added to hydroponic cultivation, SaMR12 inoculation significantly decreased MDA content in both shoot and root parts by 21–60% and 35–68%, respectively (Fig. 4b). In addition, cadmium triggered O_2_•^−^ production in the roots, which was mitigated by inoculation with SaMR12 (Fig. 5).

### Effect of SaMR12 on GSH content and proline content

**Fig. 6:**
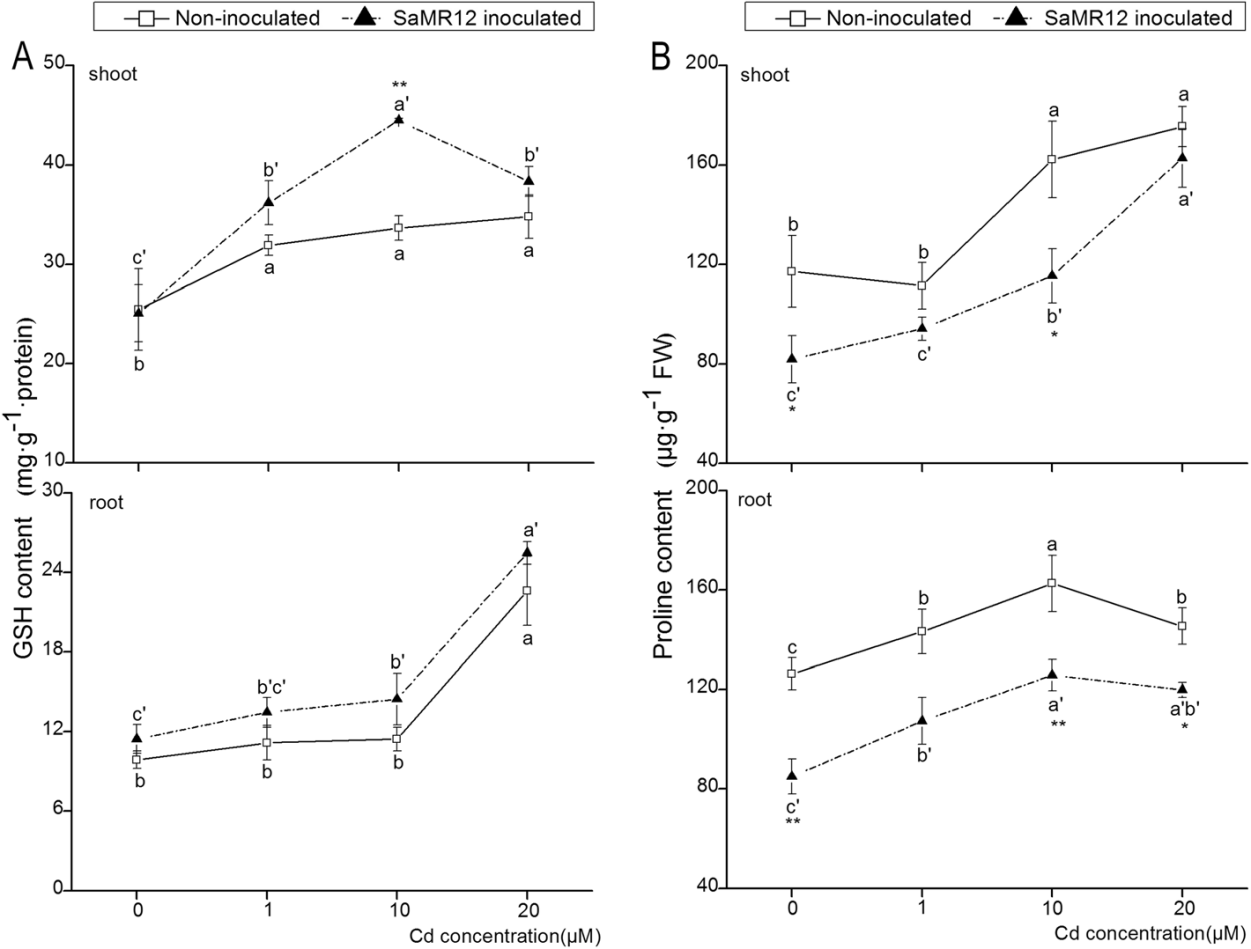
The effect of *Sphingomonas* SaMR12 on (**a**) GSH content and (**b**) proline content in both shoots and roots. The solid line, non-inoculated plants; the dashed line, SaMR12-inoculated plants. Plant samples were harvested after 7 d of treatment. The vertical line on each bar shows the standard deviation (*n* = 3). The different letters on the error bars indicate significant differences among treatments at *p* < 0.05 in the shoot and the root, respectively. The same letters above the bars indicate no difference between the treatments at *p* < 0.05. Asterisk (*) and double asterisks (**) indicate *p* < 0.05 and *p* < 0.01, respectively, for Student’s t-test carried out between SaMR12 non-inoculated and SaMR12-inoculated samples

In general, the GSH content in the shoot part was higher than that in the root part (Fig. 6a). Cd exposure led to a significant increase in GSH content in the shoot, while in the root part, a significant increase was detected only in the 20 μM Cd treatment. Inoculation with SaMR12 resulted in a significant enhancement (32%) of shoot GSH content at the 10 μM Cd treatment level. SaMR12 inoculation obviously enhanced root GSH content, but the enhancement was not statistically significant (Fig. 6a).

The proline content in the shoots gradually increased with increasing Cd levels (Fig. 6b). SaMR12 inoculation significantly decreased shoot proline content at 0 and 10 μM Cd treatment, by 30 and 28%, respectively. The root proline content increased with Cd exposure and reached a peak in the 10 μM Cd treatment. SaMR12 inoculation decreased the root proline content by 32, 25, 22 and 17%, respectively, compared to that in the non-inoculated treatments (Fig. 6b).

### Effect of SaMR12 on the activities of antioxidative enzymes

**Fig. 7:**
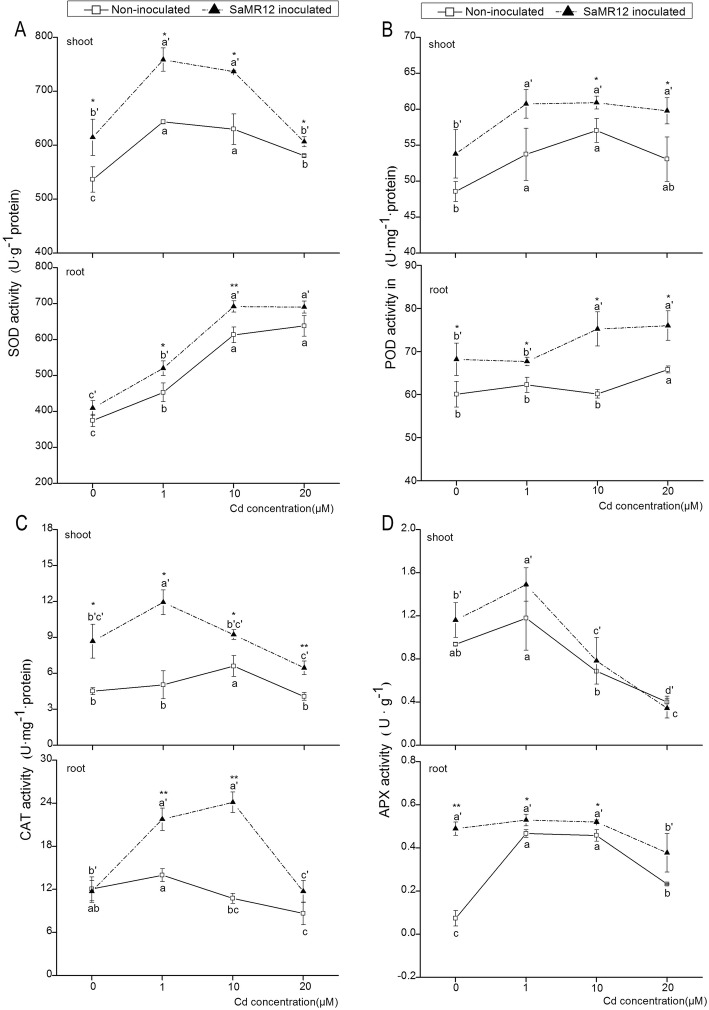
The effect of *Sphingomonas* SaMR12 on plant (**a**) SOD activity, (**b**) POD activity. (**c**) CAT activity and (**d**) APX activity in both shoots and roots. The solid line, non-inoculated plants; the dashed line, SaMR12-inoculated plants. Plant samples were harvested after 7 d of treatment. The vertical line on each bar shows the standard deviation (*n* = 3). The different letters on the error bars indicate significant differences among treatments at *p* < 0.05 in the shoot and the root, respectively. The same letters above the bars indicate no difference between the treatments at *p* < 0.05. Asterisk (*) and double asterisks (**) indicate *p* < 0.05 and *p* < 0.01, respectively, for Student’s t-test carried out between SaMR12 non-inoculated and SaMR12-inoculated samples

In the aerial parts, as the Cd concentration increased, SOD activity in the shoot first reached a peak at the 1 μM Cd treatment and then gradually decreased (Fig. 7a). With SaMR12 inoculation, shoot SOD activity was significantly enhanced at all Cd treatment levels, by 4.4–18%. However, SOD activity in the root part showed a significant increase with Cd addition and remained stable after the 10 μM Cd treatment. SaMR12 inoculation significantly activated root SOD activity in the 1 and 10 μM Cd treatments, by 15 and 13%, respectively (Fig. 7a).

Cd triggered a significant increase in POD activity in shoots, but no significant differences were detected among the different Cd treatment levels (Fig. 7b). SaMR12 inoculation significantly improved POD activity at 10 and 20 μM Cd treatments by 6.8 and 12%, respectively. POD activity in roots was higher than that in shoots under all treatments. Cd application induced a fluctuation in root POD activity, and SaMR12 inoculation significantly enhanced POD activity by 13, 8.7, 25 and 16%, respectively (Fig. 7b).

CAT activity in the roots was higher than that in the shoot parts. With Cd treatment, the CAT activity showed an increase first and then declined to its lowest value in both the aerial and underground parts (Fig. 7c). SaMR12 inoculation enhanced shoot CAT activity by 1.92-, 2.36-, 1.39- and 1.58-fold that of the non-inoculated plants, respectively. Inoculation with SaMR12 also significantly increased root CAT activity, with 0.55-fold and 1.24-fold increases at the 1- and 10-μM Cd treatment levels, respectively (Fig. 7c).

APX activity in the shoots was decreased by increasing Cd treatment levels, while root APX activity was activated at 1 μM Cd treatment but decreased under the 10 and 20 μM Cd treatments (Fig. 7d). The inoculation with SaMR12 slightly increased shoot APX activity, but the increase was not statistically significant. However, SaMR12 inoculation significantly enhanced root APX activity by 567, 13 and 13% in the 0, 1 and 10 μM Cd treatments, respectively (Fig. 7d).

### Effect of SaMR12 on gene expression levels

**Fig. 8:**
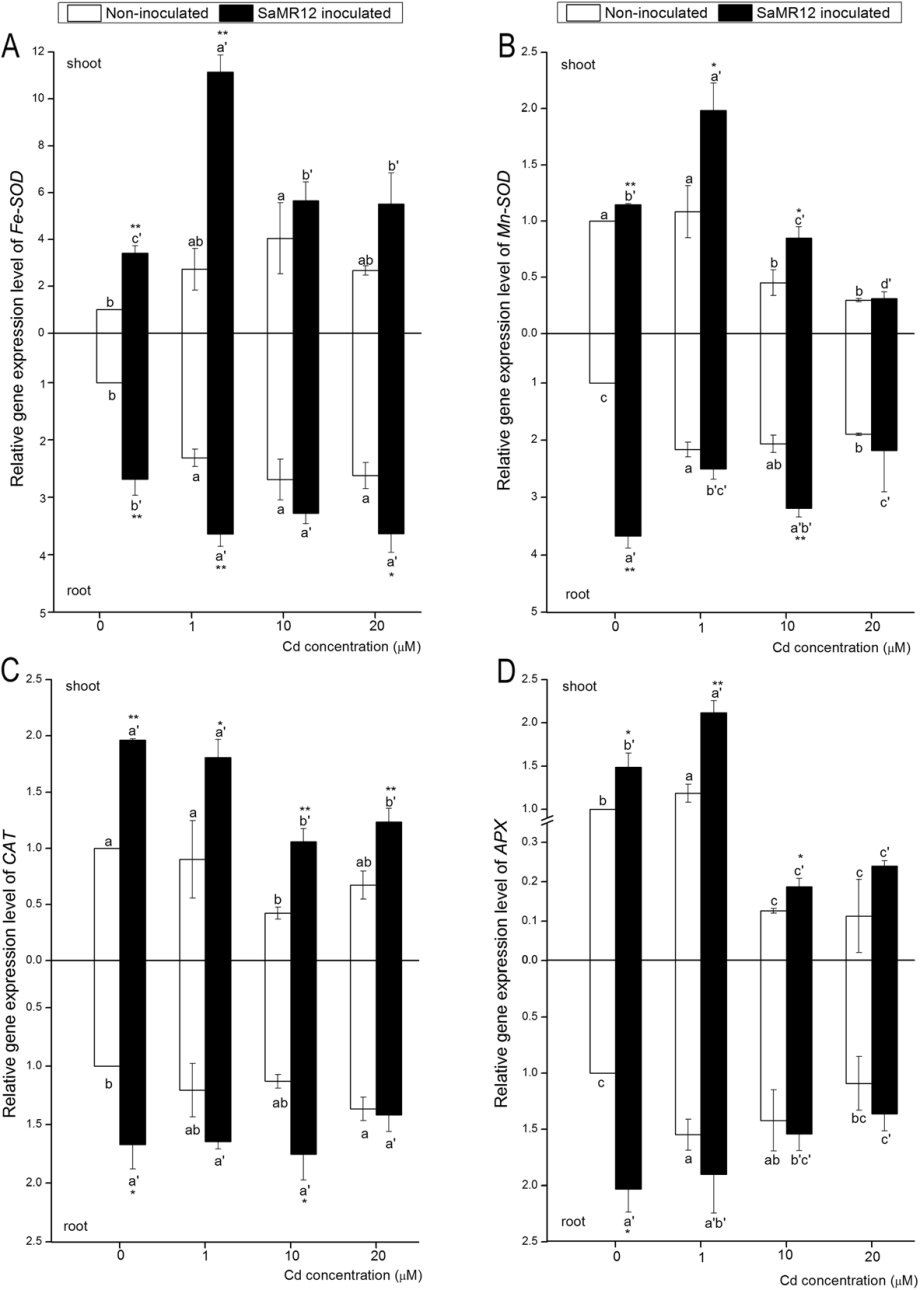
The effect of *Sphingomonas* SaMR12 on the relative gene expression levels of (**a**) *Fe-SOD*, (**b**) *Mn-SOD*, (**c**) *CAT* and (**d**) *APX* in both shoots and roots. The white column, non-inoculated plants; the black column, SaMR12-inoculated plants. Plant samples were harvested after 7 d of treatment. The vertical line on each bar shows the standard deviation (*n* = 3). The different letters on the error bars indicate significant differences among treatments at *p* < 0.05 in shoot and root, respectively. The same letters above the bars indicate no difference between the treatments at *p* < 0.05. Asterisk (*) and double asterisks (**) indicate *p* < 0.05 and *p* < 0.01, respectively, for Student’s t-test carried out between SaMR12 non-inoculated and SaMR12-inoculated samples

Antioxidant-related gene expression levels were assayed to further analyse the effect of the PGPB SaMR12 on the antioxidative system. Under Cd exposure, *Fe-SOD* expression was triggered and showed an obvious increase in both the aerial and underground parts. Inoculation with SaMR12 significantly enhanced *Fe-SOD* expression levels under 0 μM and 1 μM Cd treatment conditions, with 2.41- and 3.09-fold increases, respectively. SaMR12 inoculation also greatly enhanced root *Fe-SOD* expression levels, with 1.69-, 0.58-, 0.22- and 0.36-fold increases, respectively (Fig. 8a). Higher Cd treatment levels (10 and 20 μM Cd treatments) depressed shoot *Mn-SOD* expression levels, while SaMR12 inoculation enhanced *Mn-SOD* expression levels under the 0, 1 and 10 μM Cd treatment conditions with 0.14, 0.83 and 0.88-fold increases, respectively. Cd treatment affected root *Mn-SOD* expression, and SaMR12 inoculation significantly promoted *Mn-SOD* expression with 2.68- and 0.55-fold augmentation under the 0 and 10 μM Cd treatment conditions, respectively (Fig. 8b). Cd exposure showed very little influence on *CAT* expression levels in both aerial and underground parts. Inoculation with SaMR12 significantly improved shoot *CAT* expression with a 0.96-, 1.00-, 1.50- and 0.84-fold increase, respectively, compared to that in the non-inoculated treatments. SaMR12 inoculation also increased the *CAT* expression level in roots, but significant enhancements were observed only at the 0 μM and 10 μM Cd treatment conditions (Fig. 8c). The *APX* expression level was significantly affected by Cd exposure and reached its highest level under the 1 μM Cd treatment condition. SaMR12-inoculated plants showed a significantly higher shoot *APX* expression level in the 0, 1 and 10 μM Cd treatment conditions, with 49, 78 and 48% increases. However, the *APX* root expression level was slightly activated with Cd exposure, while SaMR12 inoculation only induced a significant increase (103%) in the 0 μM Cd treatment condition (Fig. 8d).

**Fig. 9:**
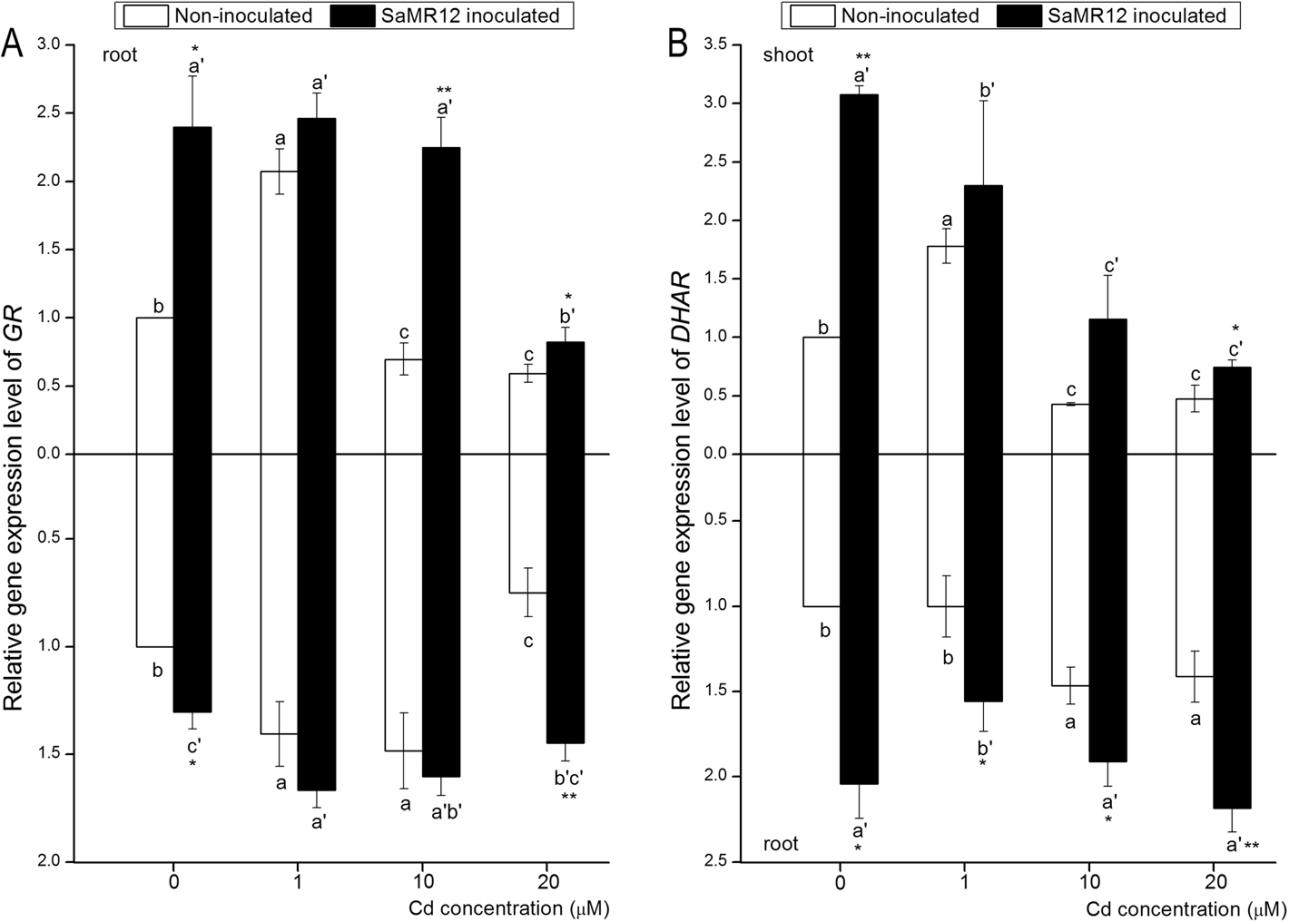
The effect of *Sphingomonas* SaMR12 on the relative gene expression levels of (**a**) *GR* and (**b**) *DHAR* in both shoots and roots. The white column, non-inoculated plants; the black column, SaMR12-inoculated plants. Plant samples were harvested after 7 d of treatment. The vertical line on each bar shows the standard deviation (*n* = 3). The different letters on the error bars indicate significant differences among treatments at *p* < 0.05 in shoot and root, respectively. The same letters above the bars indicate no difference between the treatments at *p* < 0.05. Asterisk (*) and double asterisks (**) indicate *p* < 0.05 and *p* < 0.01, respectively, for Student’s t-test carried out between SaMR12 non-inoculated and SaMR12-inoculated samples

Low concentrations of Cd showed an activating effect on *GR* expression, but higher Cd levels depressed *GR* expression (Fig. 9a). SaMR12-inoculated plants showed obviously enhanced shoot *GR* expression with a 1.40-, 0.19-, 2.22- and 0.38-fold increase, respectively, over those of the non-inoculated plants. Inoculation with SaMR12 also enhanced root *GR* expression levels. SaMR12-inoculated plants showed much higher *GR* expression in roots, with a 93% increase under 20 μM Cd treatment conditions (Fig. 9a). Different Cd treatment levels caused dehydroascorbate reductase (*DHAR*) expression changes in both the shoot and root parts. Inoculation with SaMR12 obviously enhanced shoot *DHAR* expression, and a 2.07-fold increase was detected under 0 μM Cd treatment conditions with inoculation. In roots, SaMR12 inoculation showed a significant promoting effect on *DHAR* expression levels under different Cd treatment conditions, with 1.19-, 0.56-, 0.30- and 0.45-fold increases, respectively (Fig. 9b).

## Discussion

This study investigated the colonization pattern of *Sphingomonas* SaMR12 and its effect on the growth and antioxidant system in oilseed rape growing under different Cd treatments. Root colonization of bacteria is crucial for the further expression of their beneficial effects. A previous study showed that SaMR12 could colonize plant roots such as those of its host *S. alfredii* [41], *B. napus* in pot experiments [42], and rice under hydroponic conditions [57]. The colonization pattern of SaMR12 in *S. alfredii* was reported in a detailed description by Zhang et al. [63]. The GFP-tagged SaMR12 cells first colonized and aggregated on the surface of primary roots and root junction sites and then invaded rhizodermal cells and cortical intercellular spaces of the primary and lateral roots [63]. Here, the colonization of SaMR12 on its non-host plant oilseed rape was systematically observed by CLSM at different treatment times and in different tissues. Similar to the results for *S. alfredii*, SaMR12 could successfully invade *B. juncea* during the initial 2–12 h, colonize and aggregate after 12 h in roots, gather stably at 24 h, and continue to survive at 4 d, then transfer to the shoot and colonize (Fig. 1). As shown above, the *Sphingomonas* SaMR12 could function well with high activity in oilseed rape for 4 d, which suggests that hyperaccumulator endophytes could be applied as soil remediation agents for effective phytoextraction.

It has been reported that PGPB have a promoting effect on plant growth and heavy metal accumulation [11, 12, 23, 42]. For example, inoculation with the *Pseudomonas aeruginosa* strain CPSB1 promoted wheat growth under Cd stress [47]. Inoculation with *Sphingomonas* SaMR12 promoted cadmium accumulation in *S. alfredii* [41]. In this study, SaMR12 inoculation led to an increase in plant biomass and Cd accumulation (Figs. 2 and 3), which suggested that SaMR12 could be used to promote growth and Cd accumulation in other non-host plants, such as oilseed rape. Therefore, it is a promising way to establish a plant-microbe interaction remediation system for the promotion of phytoremediation efficiency.

Cd exposure had severe deteriorating influences on the antioxidant system and resulted in lipid peroxidation through elevated levels of MDA and the generation of ROS, such as H_2_O_2_ and O_2_•^−^ [6]. The increased contents of lipid peroxides are indicators of a larger amount of toxic ROS than normal. Cd increased the *Kandelia obovata* MDA concentration [12] and the H_2_O_2_ concentration of *Sedum alfredii* Hance under hydroponic conditions [11]. This study verified that Cd induced an increase in H_2_O_2_ content, MDA content and O_2_•^−^ levels (Figs. 4 and 5), indicating that the plants suffered Cd stress and that the oxidative system was impaired. Inoculation with *Glomus intraradices* BEG 141 reduced MDA content by 33% [31]. SaMR12 inoculation lowered H_2_O_2_ concentration and MDA concentration in its host plant, *S. alfredii* [41]. Similarly, under hydroponic conditions, SaMR12 inoculation alleviated Cd-induced damage to oilseed rape by decreasing O_2_•^−^ levels, MDA content and H_2_O_2_ content (Figs. 4 and 5). These results suggest that *Sphingomonas* SaMR12 mitigated non-host plant Cd stress by reducing O_2_•^−^ levels, H_2_O_2_ content and MDA content.

In plants, proline often accumulates as a response to many biotic and abiotic stresses [9]. Proline has been reported to play a role in regulating cytoplasmic osmolytes, chelating metal ions and detoxifying ROS [56, 62]. It is reported that Cd and Pb stress caused an increase of proline content [37]. Our study showed that Cd caused an increase in proline content in both shoots and roots (Fig. 6b). However, Cd stress could be alleviated by SaMR12 inoculation, since proline content was decreased in both shoots and roots (Fig. 6b). Taken together, the results showed that *Sphingomonas* SaMR12 could alleviate Cd stress by decreasing the proline content. The results were in accordance with those of a previous study [47] in which inoculation with *Pseudomonas aeruginosa* CPSB1 reduced proline contents in Cd-treated plants. And inoculation of *Azotobacter chroococcum* also declined proline content in wheat under salt stress [51].

SOD, POD, APX and CAT are important antioxidative enzymes that help plants protect themselves against heavy metal stress. SOD has a catalysing effect on the dismutation of O_2_•^−^ to H_2_O_2_ with a remarkable reaction rate. CAT, POD and APX play important roles in scavenging H_2_O_2_ and converting it to molecular oxygen and water [16, 21]. In this study, Cd exposure increased SOD and POD activity in both shoots and roots, decreased shoot APX activity, increased CAT activity at low Cd concentrations and decreased it at higher concentrations (Fig. 7). Similar trends and opposite trends have been reported in many studies [2, 3, 12, 47, 58]. SaMR12 inoculation promoted CAT, APX, SOD and POD activities, and the subsequent qRT-PCR analysis further verified that inoculation with *Sphingomonas* SaMR12 improved *CAT, APX, Fe-SOD,* and *Mn-SOD* expression levels, especially for *Mn-SOD* and *Fe-SOD*, which showed up to 2.67-fold and 3.08-fold increases, respectively (Fig. 8). The promoted activities of CAT, APX and POD could further catalyse H_2_O_2_ into molecular oxygen and water, which explained the decrease in H_2_O_2_ content (Fig. 4). It is reported that inoculation with Cd-resistant bacteria (two *Pseudomonas* spp. and two *Rhizobium sullae*) in *Sulla coronaria* increased SOD and APX activities in both shoots and roots [14]. Inoculation with *Glomus intradices* BEG141 promoted maize SOD, POD and CAT activities under Cd exposure [31], which was consistent with our results, indicating that *Sphingomonas* SaMR12 accelerated the non-host plant response to Cd stress by increasing the activities of the enzymes of the antioxidative system, such as SOD, CAT, POD and APX.

As a key non-enzymatic antioxidant within plant cells, GSH not only provides a detoxification pathway for ROS but also participates in the regeneration of AsA via DHAR. GR catalyses the reduction of GSSG to GSH to maintain the GSH level [7, 18]. Previous studies revealed that Cd influenced the GSH-AsA antioxidant system [36] and that Cd treatment elevated GSH levels and GR levels in *S. alfredii* roots [25]. Our results showed that the GSH content and the expression levels of *GR* and *DHAR* were influenced by Cd exposure, while SaMR12 inoculation increased GSH content and promoted the expression of *GR* and *DHAR* (Fig. 6a; Fig. 9). Several other researchers have reported similar findings. For example, an increased level of GR activity in Cd-contaminated soils was observed by *Glomus mosseae* inoculation of *Cajanus cajan* [19]. Pan et al. [41] reported that inoculation with SaMR12 enhanced *S. alfredii* GSH content. *Solanum lycopersicum* L. inoculated with *Pseudomonas aeruginosa* and *Burkholderia gladioli* showed an increase in DHAR and GR without Cd but showed a decrease in DHAR and GR with Cd treatment ([29]). Elevated *GR* and *DHAR* expression led to increased GSH and AsA content, which resulted in an elevated response of the GSH-AsA cycle to Cd stress [18]. This phenomenon is consistent with the lower H_2_O_2_ increase rate (Fig. 4a). Hence, *Sphingomonas* SaMR12 could alleviate Cd stress by activating the GSH-AsA cycle, including by elevating GSH content and increasing the gene expression levels of *GR* and *DHAR*.

## Conclusion

**Fig. 10:**
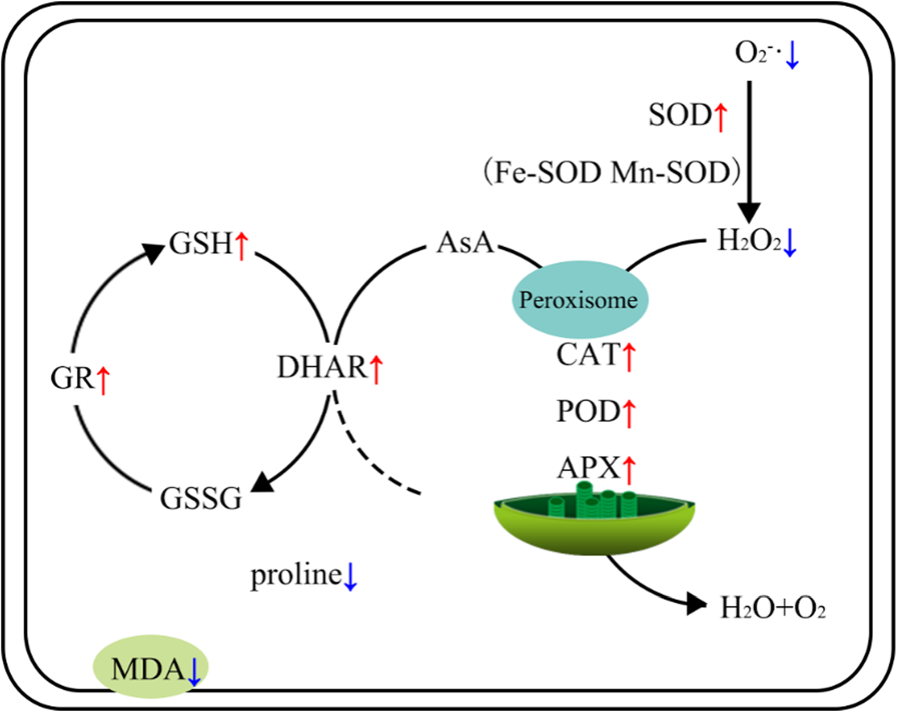
The graphical model of the effect of *Sphingomonas* SaMR12 inoculation on the antioxidative response to Cd stress in *B. juncea*. Arrows in red and blue indicate the upregulating effect and the downregulating effect, respectively. The mechanisms by which SaMR12alleviated Cd-induced abiotic stress involved 1) lipid peroxidation mitigation through reducing H_2_O_2_ content, MDA content and O_2_•^−^ levels; 2) promotion of the activities of antioxidative enzymes such as SOD, POD, CAT and APX; 3) reductions in non-enzymatic antioxidant content such as proline; and 4) activation of the GSH-AsA cycle

In the hydroponic experiment, SaMR12 successfully colonized roots at 24 h and maintained dominance in the plants for 4 d. SaMR12 inoculation promoted plant growth and increased plant Cd accumulation. SaMR12 mitigated Cd-induced abiotic stress through various pathways, including but not limited to 1) alleviating lipid peroxidation by reducing H_2_O_2_ content, MDA content and O_2_•^−^ levels; 2) increasing antioxidative enzyme activities, including those of SOD, POD, CAT and APX; 3) reducing the content of non-enzymatic antioxidants such as proline; and 4) facilitating the GSH-AsA cycle (Fig. 10). In conclusion, hyperaccumulator endophytes are proven to be effective remediation candidates that could interact with plants with greater biomass, such as oilseed rape, to increase plant tolerance to heavy metals and improve phytoextraction efficiency.

## Methods

### Plant materials and growth conditions

The *Brassica juncea* (L.) Czern. Xikouhuazi used here was a commercial extension variety, which was previously used by other researchers for experiments and identified as a mustard type of oilseed rape with high Cd accumulation [48, 53]. The seeds were purchased in 2010 at Shunda seed company in Shaoxing, Zhejiang, China (http://www.sxshunda.com/), after which seeds were propagated every year in our experimental field and stored for research. After 4.5 g agar was added to 1 L double-distilled water (ddH_2_O) and boiled, the medium was poured into a glass dish and solidified for seed germination. After washing with 70% (v/v) ethanol for 30 s followed by several rinsing steps with autoclaved ddH_2_O, seeds were sown onto the solidified medium and placed in darkness for germination. Seedlings with 3 leaves were then transferred to hydroponic conditions with 1/4 and 1/2 modified Hoagland medium in turn. The growth conditions were 22 °C with a light intensity of 180 μM m^− 2^ s^− 1^ during a 16/8 h light/dark photoperiod in a controlled growth chamber at Zhejiang University.

### Observation of the SaMR12 colonization pattern

The green fluorescence protein (GFP)-tagged *Sphingomonas* SaMR12 strains grown in Luria-Bertani (LB) liquid medium were cultured overnight at 30 °C to the exponential growth phase. Cells were collected by centrifuging at 3000×g, and cell pellets were washed twice and resuspended with autoclaved ddH_2_O to an OD_600nm_ of 1.00. The plant root inoculation was performed according to the protocols described by previous researchers with some modifications [41, 45]. The plant root systems were dipped in bacterial suspensions for 2 h and transferred to Hoagland solution. Four different sampling times were set (2 h, 12 h, 24 h and 4 d), and ten plants were randomly chosen at each sampling time to observe the SaMR12 colonization pattern using a laser scanning confocal microscope (LSCM) (ZEISS LSM780, Carl Zeiss Inc., Jena GmbH, Germany).

### Hydroponic culture and cadmium (cd) treatment

Plant cultivation was the same as described in section 5.1 until the seedlings were transplanted into 1/2 Hoagland solution. The oilseed rape seedlings were then divided into two groups. *Sphingomonas* SaMR12 was cultured as described in section 5.2, and the OD_600nm_ was adjusted to 1.00. Then, one group of plants (the inoculated plants) was soaked in the SaMR12 bacterial suspension for 2 h, while plants in another group (the non-inoculated plants) were dipped into an equal amount of ddH_2_O for 2 h. The Hoagland solution and bacterial suspensions were renewed every 3 d. Preliminary experiment was conducted to select different Cd treated levels. For determination of the short-time antioxidative response towards Cd stress, we firstly chose 1, 10 and 50 μM Cd treatment as mild, moderate and severe levels respectively. After 3 d growth however, 50 μM Cd treated level seems to be lethal to the oilseed rape (Additional file 1: Fig. S1). Thus, we adjusted to 20 μM Cd treatment. And after 7 d growth, some plants were affected by Cd treatment, but still in a good growth status. Thus, four different Cd levels (0, 1, 10, 20 μM) were applied using Cd (NO_3_)_2_·4H_2_O to both inoculated plants and non-inoculated plants with three replicates.

### Determination of plant biomass and cd content

After 30 d of treatment, plants were divided into shoots and roots, submerged in 20 mM Na_2_-EDTA for 15 min and then washed with deionized water. Afterwards, all samples were dried in an oven at 105 °C for 30 mins and then at 65 °C until completely dry. Their dry weights were recorded, and the samples were ground and passed through a 0.5 mm sieve. For Cd concentration determination, samples were digested in HNO_3_:H_2_O_2_ = 5:1 (v/v) until completely clear. After diluting with deionized water, the Cd concentration was assayed with an inductively coupled plasma mass spectrometer (ICP-MS, PlasmaQuant® MS, Germany).

Both blank and standard samples were repeated three times and digested together with the plant samples. The standard material used in this study was GBW (E) 100,351 (plant, Cd 0.42 ± 0.02 mg kg^− 1^). After determination using ICP-MS, the results showed that the Cd content of the reference material was 0.41 ± 0.015 mg kg ^− 1^.

### Determination of hydrogen peroxide (H_2_O_2_) content, superoxide radical (O_2_•^−^) levels, antioxidative enzyme activities, malondialdehyde (MDA) content, proline content and glutathione (GSH) content

After 7 d of growth, all plant samples were separated into shoots and roots, harvested and immediately stored at − 80 °C in an ultra-low temperature freezer for the following analysis. The H_2_O_2_ content and O_2_•^−^ levels in roots were measured according to the protocol described by Tian et al. [55]. The determination of antioxidative enzymes has been described in detail by Jin et al. [27]. Briefly, SOD (EC 1.15.1.1) activity was measured with the method of Giannopolitis and Ries [20] based on the photoreduction of nitro-blue tetrazolium (NBT). The reaction mixture was composed of 50 mM K-phosphate (pH 7.8), 0.1 mM EDTA, 10 mM l-methionine, 2.7 μM riboflavin and 75 μM NBT [14]. One unit (U) of SOD was recorded as the amount of enzyme that led to a 50% inhibition of NBT. CAT (EC 1.11.3.6) activity was determined by following the decline of absorbance at 240 nm caused by the decomposition of H_2_O_2_ (10 mM) in 3 min. One unit (U) of CAT activity refers to 1 nmol H_2_O_2_ dissociated in 1 min [1]. Peroxidase (POD; EC 1.11.1.7) activity was assessed with the method described by Fielding and Hall [17]. One unit (U) of POD activity refers to the amount of enzyme that oxidized 1 nmol guaiacol per minute. APX (EC 1.11.1.11) was measured by monitoring the disappearance of 0.5 mM ascorbate at 290 nm in 1 min [4]. The APX enzyme activity was calculated with an extinction coefficient of 2.8 mM^− 1^ cm^− 1^, and one unit (U) of APX activity was defined as 1 nmol ascorbate oxidized in 1 min. The MDA content was determined according to the method of Heath and Packer [24] based on its reaction with thiobarbituric acid (TBA). Fresh samples (0.2 g) were homogenized and extracted in 10 ml of 0.25% TBA, and then the extract was heated at 95 °C for 30 min and cooled on ice. After centrifuging, the absorbance of the samples was determined at 532 nm. The level of MDA was expressed as nmol mg^− 1^ protein. The proline content of samples was determined according to Bates et al. [8]. Total GSH was estimated following the method reported by Anderson [5] using a glutathione reductase-catalysed reaction. Briefly, glutathione was extracted by homogenizing 0.5 g of frozen fresh leaves in 2 mL of 5% (w/v) 5-sulfosalicylic acid. After homogenizing and centrifuging, the assay mixture was equilibrated at 30 °C for 5 min. Then, GSH-containing extract was added to each sample, after which the absorbance at 412 nm was monitored. The GSH content was determined through comparison with a standard curve of the reaction rate.

### RNA extraction and determination of the relative gene expression levels

After Cd treatment for 7 d, both the shoot parts and the root parts of seedlings were harvested, and the total RNA was isolated using an Omega RNA isolation Kit (E.Z.N.A.® Plant RNA Kit, Omega Bio-tek, Norcross, USA) according to the manufacturer’s instructions. Three biological replicates from each sample were used for this experiment. RNA concentration and quality were determined using an ultraviolet (UV) spectrophotometer (Nano-300, AS-11020-00, Hangzhou, China) and agarose gel electrophoresis, respectively.

After RNA extraction, contaminating genomic DNA was removed with RNase-free DNase (TaKaRa, Dalian, China), and the cDNA was synthesized with 1 mg of total RNA with PrimeScript RT reagent Kit (TaKaRa, Dalian, China). Quantitative real time-PCR (qRT-PCR) reactions were carried out using the SYBR Green premix Ex Taq kit (Takara, Dalian, China) on an Applied Biosystems 7900HT Fast Real-Time PCR System (ThermoFisher Scientific, Shanghai, China). All primers were synthesized according to Wu et al. [58]. qRT-PCR was performed as follows: initial denaturation at 95 °C for 30 s, then 40 cycles were set at 95 °C for 5 s, annealing at 55 °C for 30 s, and finally extension at 72 °C for 30 s. Melting curve analysis confirmed the specificity of amplification. Each qRT-PCR experiment was repeated three times. Actin was used as the internal control. The relative expression of genes was normalized by comparing with the expression of actin and analysed using the 2^−ΔΔCt^ method [32].

### Statistical analysis

One-way analysis of variance (ANOVA) was used to compare the differences among Cd treatment groups for biomass, Cd content and accumulation, antioxidative enzyme activities, antioxidant contents and gene expression levels (qRT-PCR). Statistical significance (*p* < 0.05) was analysed using Fisher’s least significant difference (LSD) test. Student’s t-test was carried out to compare the significant differences between SaMR12 non-inoculated plants and inoculated plants. All graphical work was performed with OriginPro 8.5 (Northampton, MA 01060, USA).

## Supplementary information

**Additional file 1: Figure S1.** Plant growth conditions at different Cd treated levels after 3 d. Bar = 1 cm. After 3 d growth however, 50 μM Cd treated level seems to be lethal to the oilseed rape.

## Data Availability

The datasets used and/or analysed during the current study are available from the corresponding author on reasonable request.

## Abbreviations

APX: Ascorbate peroxidase
AsA: Ascorbic acid
CAT: Catalase
Cd: Cadmium
DHAR: Dehydroascorbate reductase
GR: Glutathione reductase
GSH: Glutathione
H_2_O_2_: Hydrogen peroxide
MDA: Malondialdehyde
O_2_•^−^: Superoxide radicals
PGPB: Plant growth promoting bacteria
POD: Peroxidase
SOD: Superoxide dismutase
