# Supplementary material for: The endophytic bacterium Sphingomonas SaMR12 alleviates Cd stress in oilseed rape through regulation of the GSH-AsA cycle and antioxidative enzymes
Source: BMC Plant Biol. 2020 Feb 6;20:63. doi: 10.1186/s12870-020-2273-1 (PMC7006384; doi:10.1186/s12870-020-2273-1)
Supplement: Supplementary file 1 — Additional file 1: Figure S1. Plant growth conditions at different Cd treated levels after 3 d. Bar = 1 cm. After 3 d growth however, 50 μM Cd treated level seems to be lethal to the oilseed rape. [file 12870_2020_2273_MOESM1_ESM.docx]

**Additional file 1**


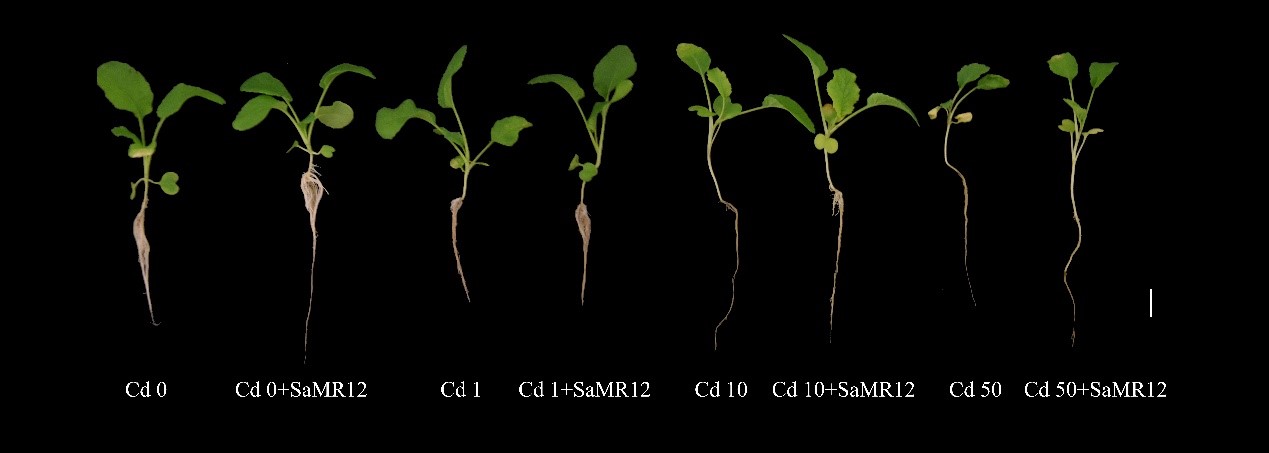


**Figure S1.** Plant growth conditions at different Cd treated levels after 3 d. Bar=1cm. After 3 d growth however, 50 μM Cd treated level seems to be lethal to the oilseed rape.
